# The kernel-weighted local polynomial regression (KwLPR) approach: an efficient, novel tool for development of QSAR/QSAAR toxicity extrapolation models

**DOI:** 10.1186/s13321-021-00484-5

**Published:** 2021-02-12

**Authors:** Agnieszka Gajewicz-Skretna, Supratik Kar, Magdalena Piotrowska, Jerzy Leszczynski

**Affiliations:** 1 Laboratory of Environmental Chemometrics, Faculty of Chemistry, University of Gdansk, Wita Stwosza 63, 80-308 Gdansk, Poland; 2 Interdisciplinary Center for Nanotoxicity, Department of Chemistry, Physics and Atmospheric Sciences, Jackson State University, 1400 J. R. Lynch Street, P. O. Box 17910, Jackson, MS 39217 USA

**Keywords:** KwLPR, QSAR, QSAAR, Risk assessment, R-script, Interspecies extrapolation

## Abstract

The ability of accurate predictions of biological response (biological activity/property/toxicity) of a given chemical makes the quantitative structure‐activity/property/toxicity relationship (QSAR/QSPR/QSTR) models unique among the in silico tools. In addition, experimental data of selected species can also be used as an independent variable along with other structural as well as physicochemical variables to predict the response for different species formulating quantitative activity–activity relationship (QAAR)/quantitative structure–activity–activity relationship (QSAAR) approach. Irrespective of the models' type, the developed model's quality, and reliability need to be checked through multiple classical stringent validation metrics. Among the validation metrics, error-based metrics are more significant as the basic idea of a good predictive model is to improve the predictions' quality by lowering the predicted residuals for new query compounds. Following the concept, we have checked the predictive quality of the QSAR and QSAAR models employing kernel-weighted local polynomial regression (KwLPR) approach over the traditional linear and non-linear regression-based approaches tools such as multiple linear regression (MLR) and *k* nearest neighbors (*k*NN). Five datasets which were previously modeled using linear and non-linear regression method were considered to implement the KwPLR approach, followed by comparison of their validation metrics outcomes. For all five cases, the KwLPR based models reported better results over the traditional approaches. The present study's focus is not to develop a better or improved QSAR/QSAAR model over the previous ones, but to demonstrate the advantage, prediction power, and reliability of the KwLPR algorithm and establishing it as a novel, powerful cheminformatic tool. To facilitate the use of the KwLPR algorithm for QSAR/QSPR/QSTR/QSAAR modeling, the authors provide an in-house developed *KwLPR.RMD* script under the open-source *R* programming language. 
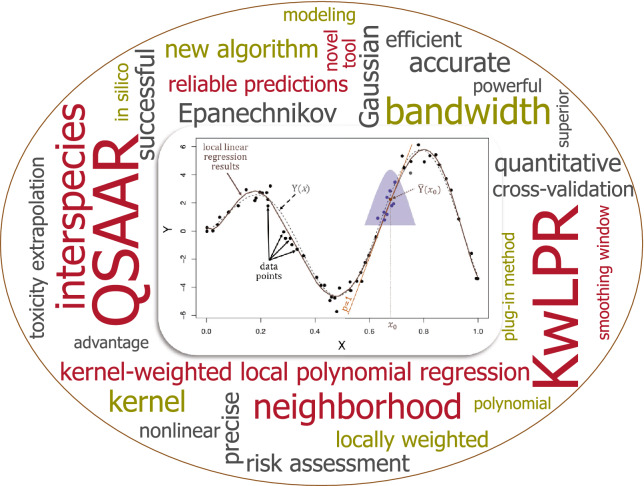

## Introduction

The biological response, physicochemical properties as well as intrinsic toxicity of a chemical have a strong relationship with its structural representation. This can be mathematically modeled with a series of chemical information employing quantitative structure–activity/property/toxicity relationship (QSAR/QSPR/QSTR) and/or read-across models [1]. Over the last two decades, the QSAR models became an integral part of computer-aided drug design (CADD) and discovery [2, 3], earlier prediction of adsorption, distribution, metabolism, excretion, and toxicity (ADMET) of new drug candidates [4, 5], environmental risk assessment through fate and toxicity modeling of chemicals [6, 7], solution of diverse complications in materials sciences [8, 9], food science [10] along with agricultural science [11]. Researchers from academia and industries employ QSAR models as a popular technique for data gap filling through early prediction of activity and toxicity of various chemicals and pharmaceuticals even before their synthesis. Rigorously validated and statistically significant QSAR models may significantly help the prioritization strategy for future synthesis and analysis, leading to substantial advantages in saving resources in the form of materials, human resources, time, and money [12].

The interspecies quantitative activity–activity relationship (QAAR)/quantitative structure–activity–activity relationship (QSAAR) model offers to predict an endpoint (which is a dependent variable) for specific species employing the same endpoint (response in the form of activity, property, or toxicity) for another species along with selected structural and physicochemical features as a predictor or explanatory or independent variables (descriptors) [13, 14]. The terms QAAR and QSAAR are used interchangeably by different groups of authors, but for the ease of understanding and concept of the present study, we will use the term QSAAR throughout the manuscript. The endpoint acts as a predictor variable, it can highlight the mechanism of action (MOA) of a series of chemicals to some extent as they are derived from experimental bioassay along with structural and physicochemical features. This specific feature strengthens the reliability and precision of the QSAAR model over the simple QSAR models. Furthermore, when experimental data of a series of chemicals for one species is present but absent for another species, the QSAAR model delivers a mathematical model (or equations) to predict the endpoint for that specific species [15]. Thus, extrapolating data from one species to another helps fill the data gaps without wasting time, money, and animal study maintaining the 3R's approach intended to a replacement, reduction, and refinement of animals.

The most common techniques for developing QSAR and QSAAR models are Multiple Linear Regression (MLR), Principal Component Regression (PCR), Partial Least Squares (PLS), under linear regression technique [1–3]. While, *k* nearest neighbors (*k*NN), artificial neutral network (ANN), support vector machine (SVM) are the most frequently practiced techniques for non-linear regression modeling. Once the model is developed, it requires to be checked through rigorous validation methods (cross-validation, test set validation, Y-randomization) using stringent validation metrics (R^2^, Q^2^_LOO_, R^2^_pred_/Q^2^_F1_, Q^2^_F2_, Q^2^_F3_, r_m_^2^, CCC, mean absolute prediction error (MAE), Root Mean Square Error (RMSE)) [1]. The applicability domain strategy is useful to identify those chemicals that cannot be predicted reliably by the model. Most importantly, majority of models offer excellent performance for closely related chemicals structure-wise while the prediction error can be in the higher side for the new query chemical which is located outside of the AD generated from the training set [2, 3].

To reinforce the scientific and systematic validity of any QSAR/QSAAR model and promote its acceptance for regulatory purposes, drug design and discovery, toxicity prediction to humans and the environment, the correct statistical approach for developing the model is an imperative one. Over the years, multiple forms of linear, polynomial, lasso, ridge, ecologic, Bayesian, ElasticNet, etc*.* regression approaches have been evolved [16]. Without any doubt, the most widely used and acceptable form of regression has been linear regression. Still, drawbacks like over-fitting and sensitivity to both cross-correlations and outliers are a matter of concern. In comparison, ridge and lasso represent a more vigorous version of linear regression taking constraints on regression and being less subject to over-fitting and straightforward interpretation. In polynomial regression, the coefficient can be changed with the predictor or explanatory variable's value and estimated from data that lie within a specific window. The polynomial regression is suitable to evaluate the density of distribution and can be successfully employed when there are two or more predictor variables present in the model [17]. The polynomial models are also instrumental where the relationship between response study and predictor variables is curvilinear. Additionally, a nonlinear relationship in a narrow range of explanatory variables can also be modeled by polynomial regressions.

The use of the kernel-based nonparametric approach to regression analysis has a long tradition in econometrics. Its application in computational toxicology and chemical risk assessment has a much shorter history. Although multiple examples of traditional QSAR/QSPR models based on nonparametric regression exist [18, 19], the attempt to employ the proposed approach to interspecies QSAAR modelling has not been presented in literature before, to the best of the authors' knowledge. Hence to introspect the advantages and predictive quality of the kernel-weighted local polynomial regression (KwLPR) approach over the traditional linear and non-linear regression-based methods we have re-developed both QSAR and QSAAR models. Therefore, we have taken five different datasets of varying sizes from big, medium to small, and used diverse chemical classes of compounds for modeling purposes. Four datasets [20–22] were previously utilized to develop the high-quality QSAAR model using a common linear regression technique like MLR and one dataset [23] was employed to develop non-linear model implicating *k*NN technique. In present study, all five datasets were used to develop models utilizing the KwLPR approach. Then we have compared the statistical quality of our models with the previous models. It is important to mention that we have used the same modeled descriptors and the same combination of training and validation sets compounds as was done in the original papers. This study's idea is neither the generation of the new prediction oriented QSAR/QSAAR models nor criticizing the previous ones. The present KwLPR models demonstrate the worth of the locally weighted least squares kernel regression in interspecies extrapolation as well as in application to simple QSAR model by offering better statistical quality than previously developed models. The R-script code to prepare the KwPLR based QSAR/QSPR/QSTR/QSAAR model is also provided for better accessibility.

## Materials and methods

The main idea behind the locally weighted least squares kernel regression that combines the mathematical simplicity and interpretability of the classical least squares method with the flexibility of nonlinear regression is the pointwise approximation of the unknown regression function *m(x)* by a polynomial of order *p* in a small neighborhood of *x*_*0*_ [24]. Using a local Taylor series expansion in the neighborhood of *x*_*0*_, a *p*^*th*^ degree polynomial approximation of *m(x)* yields [25]:

$$m\left(x\right) \approx \sum_{j=0}^{p}\frac{{m}^{\left(j\right)}{(x}_{0})}{j!} {\left(x-{x}_{0}\right)}^{j} \equiv \sum_{j=0}^{p}{\beta }_{j}{\left(x-{x}_{0}\right)}^{j}$$

This polynomial is fitted locally at each point of interest by weighted least squares (also termed as kernel-weighted linear regression), that minimizes:

$$ \mathop {{\text{min}}}\limits_{{\beta _{j} }} \sum _{{{\text{i}} = 1}}^{{\text{n}}} \left\{ {\underbrace {{Y_{i}  - \sum\limits_{{j = 0}}^{p} {\beta _{j} } \left( {x_{0} } \right)(x_{i}  - x_{0} )^{j} }}_{{polynomial}}} \right\}^{2} \underbrace {{K\left( {\frac{{x_{i}  - x_{0} }}{h}} \right)}}_{{local}} $$

where: *X* denotes the design matrix centered at *x*_*0*_;* Y* represents a vector with the response variable; *β* is a vector of regression coefficients obtained by applying weighted least squares; *K* denotes a non-negative kernel function assigning weights to each point; *h* is a smoothing parameter controlling the size of the local neighborhood; whereas *n* is the number of independent variables.

Using matrix notation, one can write this as [25]:

$$\underset{\beta }{\mathrm{min}}\left\{{\left(y-X\beta \right)}^{T}W(y-X\beta )\right\}$$

where:

$$y={({Y}_{1}, {Y}_{2},\dots \dots , {Y}_{n})}^{T}$$

$$X=\left(\begin{array}{c}\begin{array}{c}\begin{array}{c}1 \left({x}_{1}-{x}_{0}\right) \cdots {\left({x}_{1}-{x}_{0}\right)}^{p}\\ 1 \left({x}_{2}-{x}_{0}\right) \cdots {\left({x}_{2}-{x}_{0}\right)}^{p}\end{array}\\ \vdots \vdots \vdots \end{array}\\ 1 \left({x}_{n}-{x}_{0}\right) \cdots {\left({x}_{n}-{x}_{0}\right)}^{p}\end{array}\right)$$

$$\beta ={({\beta }_{0}, {\beta }_{1},\dots \dots , {\beta }_{p})}^{T}$$

$$W=diag\left\{K\left(\frac{{x}_{i}-{x}_{0}}{h}\right)\right\}$$

The solution vector of coefficients *β* is provided by weighted least squares theory and can be conveniently expressed as [25]:

$$\widehat{\beta }= {({X}^{T}WX)}^{-1}{X}^{T}Wy$$

where: *W* represents a diagonal matrix of weights.

**Fig. 1:**
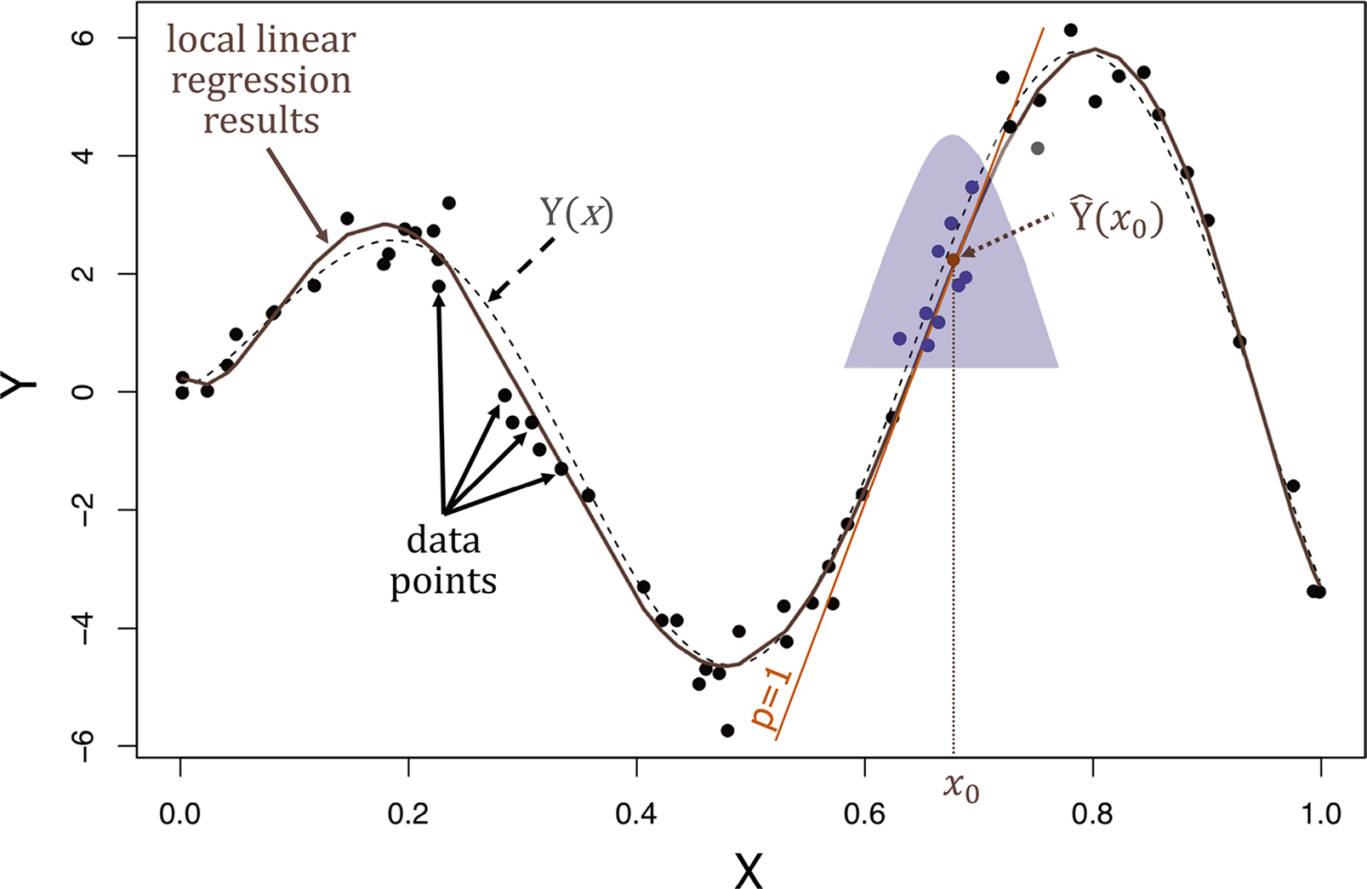
Locally weighted least squares kernel regression is illustrated with simulated data, where the dashed grey curve represents *m(x)* from which the data were generated, while the solid brown curve corresponds to the locally weighted linear regression estimate. The purple-colored points are the neighboring points to the query point whose response is estimated (*x*_*0*_). The light purple bell-shape superimposed on the plot indicates weights assigned to the adjacent points, decreasing to zero with increasing distance from the query point

In the light of the above, the main advantage of the kernel-weighted local polynomial regression approach is that unlike the most common approaches for regression analysis, applied in QSAR/QSAAR studies (e.g. LR, MLR, PCR, PLS, etc*.*), where the regression coefficients are estimated using the least squares method by minimizing the sum of the squared residuals on the training data, it employs only a small batch of training data from the entire training set, that are most chemically similar to a given target point. This means that in the local polynomial regression approach, the regression coefficients are estimated with a sliding smoothing window [*x*_*0*_*—h(x*_*0*_*)*, *x*_*0*_ + *h(x*_*0*_*)*] by fitting a polynomial of degree *p* locally at each query point (Fig. 1). Only observations within that smoothing window are used to approximate the unknown regression function *m(x)* by a polynomial, whereas the coefficients of this polynomial are fitted at each point of interest by weighted least squares regression [24]. It is worth emphasizing that the shape and width of the smoothing window, that is, in practice the shape and extent of the local regression neighborhood is determined by the kernel function and bandwidth described below:

1. Kernel function (*K*) specifies the neighborhood's shape and assigns weights to the neighboring points based on the distance to the target point. In the overall weighting scheme, the most significant weights are given to data points closest to the target point whose response is being estimated than those that are further away. So simply speaking, the weights determine how much each response value of the neighboring training data points influences the activity of a given fitting point.
  $$\mathrm{K}\left(\frac{{\mathrm{x}}_{\mathrm{i}}-{\mathrm{x}}_{0}}{\mathrm{h}}\right)$$
2. Bandwidth also called the smoothing parameter (*h*) dictates the width of the kernel function (i.e., the width of the neighborhood). In practice, bandwidth and kernel function determine the number of nearest neighbors for regression.

As discussed elsewhere [26], the most relevant factors affecting the statistical properties of kernel-weighted local polynomial regression are the degree of the polynomials (*p*), the bandwidth (*h*), and the chosen kernel function (*K*). A brief description of the above-mentioned parameters is given below.

### Degree of local polynomials

**Fig. 2:**
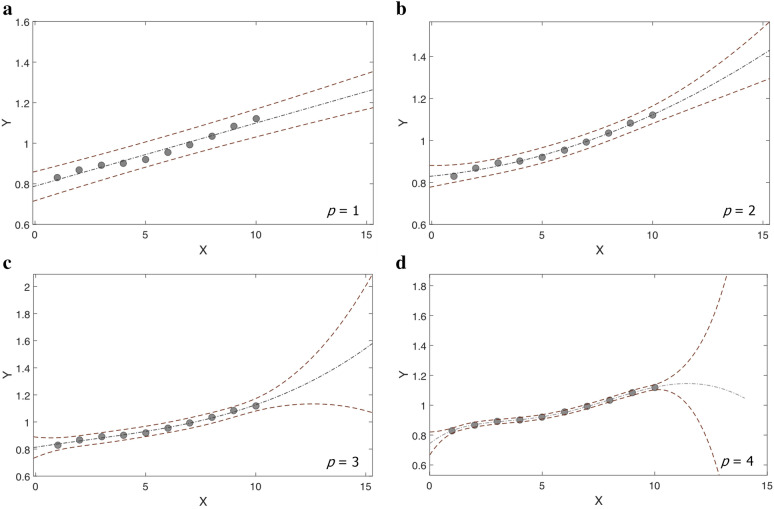
Schematic representations of extrapolation at various polynomial’s orders are illustrated using simulated data. The polynomials order ranges from 1 (**a**) to 4 (**d**). The brown dashed lines represent the 95% confidence interval limits

The benefits, drawbacks, limitations, and applicability of different local polynomial degrees have been extensively reviewed in the literature [24, 26]. Generally, as the flexibility in the model fitting increases (by increasing the degree of the polynomial used), the local-polynomial estimator's bias declines, but at the same time, the variance increases. Thus, high-degree polynomials will tend to overfit the data. The choice of degree of the approximating polynomial should appropriately balance the trade-off between bias and variance. It is fairly evident that in a relatively flat (non-sloping) region, a local constant (*p* = 0) or local linear (*p* = 1) estimator is preferred. In contrast, at peaks and valleys, the most common choices are the local quadratic (*p* = 2) and local cubic (*p* = 3) estimators [27]. In practice, for spatially inhomogeneous curves, an order of polynomial approximation is adjusted to the curvature of the unknown regression function *m(x)* in the fixed neighborhood of *x*_*0*_ by choosing that order *p* for which the estimated mean squared error (MSE) of the estimator is the smallest. Additionally, as Ruppert and Wand [28], and Fan and Gijbels [26] pointed out, good practice in local polynomial regression is to adopt low odd-degree local polynomials since they have a more straightforward asymptotic bias expression. However, an in-depth review of the literature shows, in practice, the constant, linear, and quadratic polynomials (*p* ≤ 2) are the most frequently used. An additional motivation behind using the low-order polynomials is that they appear to provide an adequate prediction when extrapolated beyond the range of the given data [29]. Although the extrapolation in QSAR/QSAAR predictions is more tenuous than the interpolation, it is a common practice. In general, the estimates based on low-order polynomial extrapolation, similarly to linear extrapolation, are capable of reasonable approximation, especially when the region of extrapolation is not too far beyond the data range. However, one should be aware that this will not hold true for high-order polynomials (Fig. 2). High-order polynomials frequently fail and result in grossly misleading predictions. Hahn [29] and others [30, 31] stressed the inadequacy of high-order polynomials for extrapolation.

### Bandwidth

**Fig. 3:**
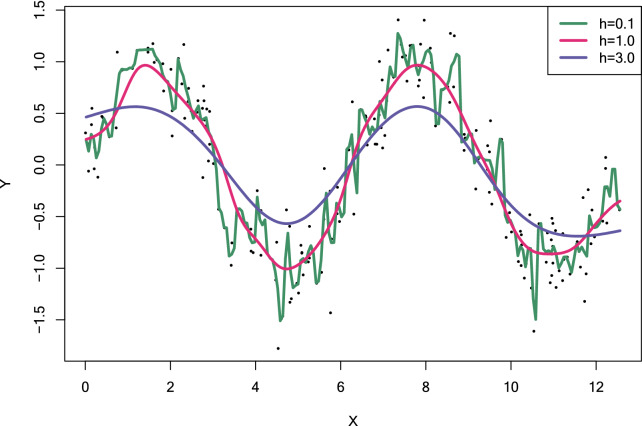
The effects of changing the smoothing parameter values are illustrated with simulated data. It is straightforward to see that small bandwidth value (the dark green curve) corresponds to large variability and small bias, whereas small variability for the highest bandwidth value (purple curve) results in large bias

The bandwidth governs the complexity of the model, and therefore the choice of the smoothing parameter is of crucial importance for every kernel regression [32]. A broad bandwidth tends to over-smooth the data with a large bias (i.e., resulting in underfitting), whereas a small bandwidth on the contrary, greatly restricts neighborhood size, and produces to a high-variance estimate (i.e., resulting in overfitting) (Fig. 3) [27].

Thus, to attain the trade-off between the goodness-of-fit and the model complexity, bandwidth should be optimized in the kernel-based regression methods. To this date, two main strategies have been devised for the bandwidth selection, namely: constant bandwidth selection and variable bandwidth selection [33]. As the name suggests, a constant bandwidth is constant across the entire range of the data (i.e., for all *x* in *m(x)*). In general, it is a reasonable choice when the unknown curve is spatially homogeneous. However, it might not be best suited to capture the unknown regression function's complexity that shows different behavior in different regions. Hence, for estimating coefficient functions with a more complicated shape of the curve, it may be desirable to use a bandwidth that varies according to the fitting point *x* at which *m(x)* is estimated. This bandwidth is referred to as local variable bandwidth and is denoted by *h(x)*. Despite a vast number of bandwidth selection techniques, most of these methods are based on minimizing the mean squared error (MSE) or the mean integrated squared error (MISE). Among the automatic data-driven bandwidth selection procedures, the most commonly used are, e.g. direct plug-in method [34], cross-validated bandwidth method, least-squares cross-validation method [35], smoothed cross-validation method [36], and the contrast method [37]. An interesting comprehensive review of bandwidth selection techniques and their applications can be found in [27, 38–40].

### Kernel function

**Fig. 4:**
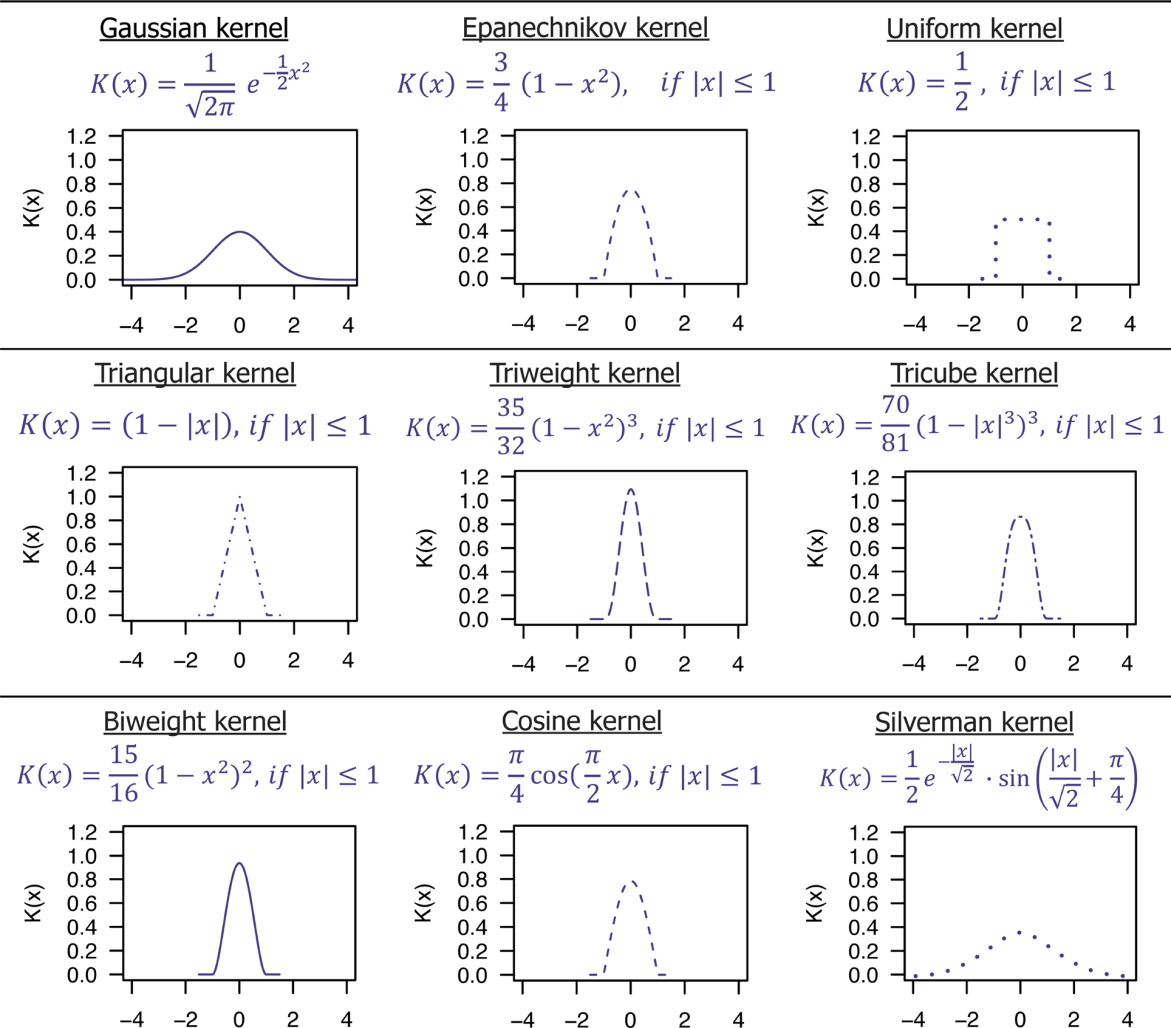
Kernel shapes and expressions

As has already been pointed out, the kernel's choice determines the local neighborhood's shape over which the smoothing is performed. Different kernels merely vary in the relative weights assigned to points closer/farther in relation to a regression point. However, the particular form of the function, *K* has only a relatively small effect on estimation accuracy. Therefore, the differentiable kernels with low computational complexity such as the Gaussian kernel or Epanechnikov kernel are being favored. To date, several kernel functions have been proposed, the most common are plotted in Fig. 4.

### Datasets

As a proof of concept, the kernel-weighted local polynomial regression approach has been applied to five diverse data sets, differing in both numbers and types of chemicals. A summary of the selected datasets that were previously used to develop ecotoxicity QSAR/QSAAR models using linear and non-linear regressions, along with the quality metrics provided by the authors of the original contributions, is given in Table 1. However, it should be highlighted that the specific focus of this study is neither the development of the new prediction oriented QSAR/QSAAR models nor criticizing the existing ones. The cited models serve as illustrative case studies to demonstrate the usefulness of the locally weighted least squares kernel regression in simple as well as interspecies toxicity extrapolation. In order to make a straightforward comparison between initially employed approaches for QSAR/QSAAR model development and the proposed KwLPR approach, the same training and validation sets, as well as the same independent variables (as in original works), have been used (Additional files 1, 2, 3, 4, 5).

**Table 1:** The details of the previous QSAR/QSAAR models employed as case studies for the present work

No. case study		Group of chemicals	Model	Refs.
Case study 1		Pesticides	MLR technique	[20]
pLC_50_ (*O. mykiss*) = 0.27 + 0.17 Log P + 0.67 pEC_50_ (*D. magna*) n_T_ = 254; R^2^ = 0.813; RMSE_C_ = 0.65; F = 545.77; p < 0.00 n_V_ = 64; RMSE_P_ = 0.68; Q^2^_F1_ = 0.817; Q^2^_F2_ = 0.817; Q^2^_F3_ = 0. 794; CCC = 0.894				
Case study 2		Pesticides	MLR technique	[20]
pLC_50_ (*L. macrochirus*) = 0.09 + 0.18 Log P + 0.67 pEC_50_ (*D. magna*) n_T_ = 235; R^2^ = 0.831; RMSE_C_ = 0.65; F = 570.92; p < 0.00 n_V_ = 59; RMSE_P_ = 0.68; Q^2^_F1_ = 0.831; Q^2^_F2_ = 0.831; Q^2^_F3_ = 0. 818; CCC = 0.900				
Case study 3		Pharmaceuticals and personal care products (PPCPs)	MLR technique	[21]
pEC_50_ (*O. mykiss*) = 1.31 + 1.24 pEC_50_ (*D. magna*) – 0.36 GATS1e n_T_ = 35; R^2^ = 0.91; RMSE_C_ = 0.45; Q^2^_LOO_ = 0.89; n_V_ = 15; Q^2^_Ext_ = 0.77–0.77; RMSE_P_ = 0.71; CCC = 0.89				
Case study 4		Substituted phenols	LR technique	[22]
pT (*C. vulgaris*) = 0.72 pT (*T. pyriformis*) + 0.25 n_T_ = 31; R^2^ = 0.75; Q^2^_LOO_ = 0.72; RMSE_C_ = 0.32 n_V_ = 10; Q^2^_Ext_ = 0.81–0.82; RMSE_P_ = 0.28				
Case study 5		Organic chemicals	*k*NN technique	[23]
pLC_50_ (*P. promelas*) = *f*(MLOGP; CIC0; SM1_Dz(Z); GATS1i; NdsCH; NdssC) n_T_ = 726; *k* = 6; R^2^ = 0.62; RMSE_C_ = 0.879; Q^2^_CV_ = 0.61; RMSE_CV_ = 0.878; n_V_ = 182; Q^2^_EXT_ = 0.61; RMSE_EXT_ = 0.888				

### Interpretability of the KwLPR model

As the proposed KwLPR modelling method does not provide a single model equation that would allow to quantify the relative importance of individual explanatory variables on the endpoint of interest, it can be hastily perceived, therefore, as unremarkable. This inherent limitation can be easily overcome. Hence, to gain mechanistic insight into the nature of the interrelationships among the modeled activity and the related descriptors, the factor loadings derived from principal components analysis (PCA) should be evaluated. In essence, the loadings refer to the correlation coefficients between original variables and the particular principal component (PC), and thereby indicate the strength and direction of a linear association. A positive loading implies that a given descriptor correlates positively with the PC, whereas a negative loading means an inverse correlation. In order to enhance the readability and the interpretability of the KwLPR model, the PCA biplot was used. This choice was motivated by the fact that the PCA biplot simultaneously shows both the observations and the variables as well as provides information on the strength of the relationship (expressed by the vector length) and the degree of correlation among the variables (expressed by the angles between the loading vectors: an adjacent angle implies high positive correlation; a straight angle indicates high negative correlation, whereas a right angle suggests no correlation between two variables).

### R-script

To facilitate the use of the kernel-weighted local polynomial regression approach for QSAR/QSAAR modeling, we additionally provide an in-house developed script as Supplementary information (Additional file 6). The script is all written in the open-source *R* programming language [41], and it heavily relies on the '*np*' package [42]. An excellent and detailed introduction to '*np*' package is provided in Hayfield and Racine publication [43]. To make the above-mentioned code as much user-friendly tool as possible and to provide its further functionality (i.e. preview plots and outputs all in a workspace) it is written in a. RMD file (i.e. *R* Markdown file created using *RStudio* as a graphical front-end to *R*). *KwLPR*.*RMD* script requires a single input data matrix written into a. CSV file, placed in the same working directory as the source *R*-code file (to see an example of an input data file please refer to the Supplementary information). The final modeling outputs are two-fold: (i) a single summary table with the most informative quality metrics organized by kernel regression estimator, degree of local polynomials, and kernel functions; and (ii) detailed output files for every single possible combination of the most influential and frequently used modeling parameters that might affect the quality of the fit. A summary table into a. CSV file is saved in the current working directory where the input file, as well as the source R-code file, are located. Whereas, detailed output files for each individual modelling scheme (including computational results, plot of model predicted and experimentally observed endpoint of interest) are saved in the automatically created nested subdirectory of the working directory.

Noteworthily, aside from comprehensive model development and its validation, initial data transformation that ensures that all variables receive equal attention during the training process is a crucial step, which cannot be overlooked. This is extremely important, since different variables mostly span several orders of magnitude and/or different ranges of units, whereas those with large numerical values can compromise the stability and statistical validity of any predictive model. To alleviate this problem, the script offers automatic data transformation also known as auto-scaling. As previously mentioned, *KwLPR*.*RMD* code intends to facilitate the kernel-weighted local polynomial regression modeling using the most commonly used bandwidth selection methods, kernel regression estimator as well as kernel functions. Its current version uses two implemented automatic data-driven bandwidth selection procedures, namely: expected Kullback–Leibler cross-validation (cv.aic), and least-squares cross-validation (cv.ls). Moreover, the current implementation of the *KwLPR*.*RMD* script permits application of two different degrees of the local polynomial smoother, namely: local-constant (Nadaraya-Watson) estimator (lc), and local-linear estimator (ll). Besides, it contains three of the most popular kernel functions, i.e.: Gaussian, Epanechnikov, and uniform. It is fairly obvious that although the use of the provided script does not require expertise and/or advanced knowledge of *R*, however, users who are more familiar with *R* language can easily customize this code further as per their requirements, by employing, for example, different bandwidth selection method and/or kernel function than is proposed herein. It should be emphasized, moreover, that the provided *KwLPR*.*RMD* script can also be successfully applied for the development of any traditional QSAR/QSPR/QSTR/QSAAR model.

## Results and discussion

To address the aim of this study and to introspect the advantages and predictive quality of the KwPLR approach over the traditional linear and non-linear regression-based methods, we have employed five toxicity datasets that were previously utilized to develop the QSAR/QSAAR models. To keep the modeling strategy as consistent as possible, the following rules have been maintained:

- The division of training and validation sets are the same (as in original works) for all five datasets.
- The modeled descriptors are also identical compare to the previously reported models.
- We have computed all classical internal and external validation metrics to quantify each QSAR/QSAAR model's quality.
- We have used the KwLPR approach to develop the QSAR/QSAAR model for all five datasets, while previously MLR, MLR, MLR, LR and *k*NN techniques were used.
- In all four QSAAR models, we have modeled higher taxonomic class species using the response value of lower taxonomic class species along with other structural and physicochemical features. Most of the QSAAR models aim to fill up the response data gap by extrapolating data. It is always practical and reasonable to extrapolate response data from lower taxonomic species to the higher one.

### Case study 1

**Fig. 5:**
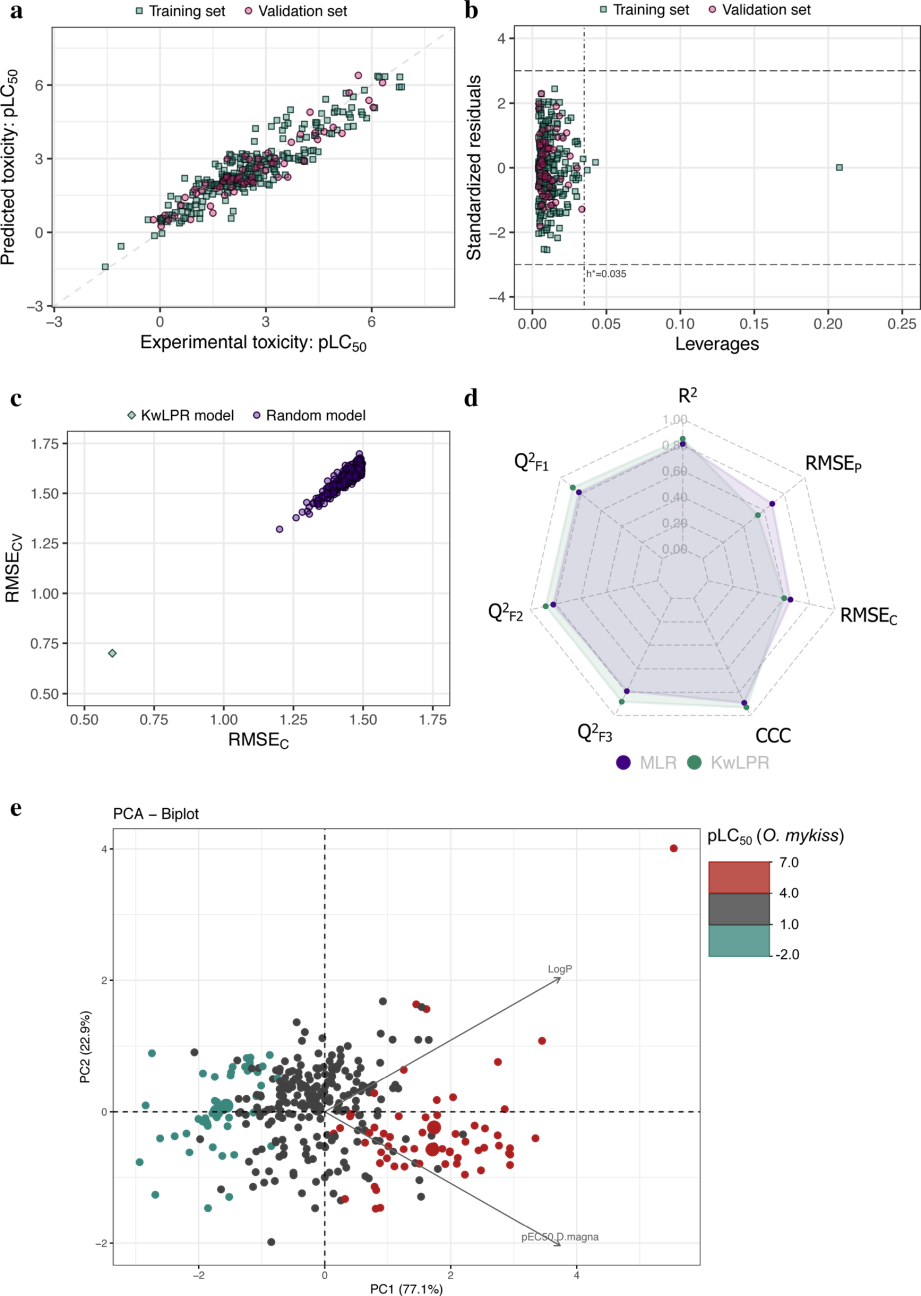
**a** Scatter plot of experimentally determined versus predicted values of pLC_50_. The formed straight line represents perfect agreement between the observed and calculated values. **b** Williams plot illustrating the applicability domain of the KwLPR model. **c** The results of the 500-fold Y-scrambling test. **d** Spider plot presenting the comparison of KwLPR model statistics with the MLR modeling approach. **e** PCA biplot

The complete dataset consists of 318 pesticides with quantitative toxicity value in the form of LC_50_ to *O. mykiss* and *D. magna*. The toxicity values covered the toxicity range of 8.398 logarithmic units. The authors of the original contribution reported a two-descriptor QSAAR model obtained through MLR for determining the toxicity of 318 pesticides to *O. mykiss* [20]. The model calibration was performed by using 254 pesticides as a training set, while external validation was carried out by using 64 compounds as a test set. The logarithmic value of the partition coefficient (Log P) and experimental toxicity value of *D. magna* (pEC50) were used as independent variables. The previous model reported a determination coefficient (R^2^) of 0.813, while the present model using KwLPR increases this value to 0.85 using the same modeled descriptors. Similarly, all three external correlation validation metrics showed improved value for the KwLPR based QSAAR model (Q^2^_F1_ = 0.88; Q^2^_F2_ = 0.88; Q^2^_F3_ = 0.88 while previous one had 0.817, 0.817 and 0.794, respectively). A similar trend is observed for the CCC parameter. The present model is also less error-prone than the previous one which is reflected in the lowest value of the RMSE in both training and validation sets (RMSE_C_ = 0.60; RMSE_P_ = 0.54 while previous one had 0.65 and 0.68, respectively). For detailed information on the experimental and predicted toxicity data for particular compounds as well as the numerical values of the molecular descriptors used within this study, please refer to the Supplementary information (Additional file 7: Table S1). The scatter plot (Fig. 5a) of experimental and predicted toxicity values illustrated that training and validation set chemicals scatter on both sides of the line of the perfect fit, and no points have deviated within ± 1 value. The Williams plot (Fig. 5b) for the applicability domain (AD) analysis suggested no validation compounds are outliers. In contrast, two training compounds showed higher leverage value compare to the critical value (h^*^) of 0.035. Both compounds behave as influential observations (X outlier), although they were not response outliers (not Y outlier). We have also performed 500-fold Y-scrambling test where the plot in Fig. 5c suggested the KwPLR model was not obtained by chance and the model is extremely reliable. The Spider plot (Fig. 5d) suggested that KwPLR has superior regression-based (higher and close to 1), as well as error based metrics (lower and close to 0) values, compared to MLR implemented in the previous model. To provide an insight into the structure–activity relationship of the studied pesticides the PCA analysis was performed (Fig. 5e). It is straightforward to see that pLC50 (*O. mykiss*) increases moving from left to right along the X-axis, termed as first principal component (PC1). Due to low PC1 scores and positive loading values, the pesticides with lower values of pLC50 are characterized by relatively lower lipophilicity (LogP) and toxicity to *D. magna* (pEC50 [mM]) compared to pesticides with higher values of pLC50. An acute angle between both variables indicates moderate positive correlation (r = 0.54), whereas comparable vectors length indicates that both variables equally contribute to describe the toxicity towards *O. mykiss*.

### Case study 2

**Fig. 6:**
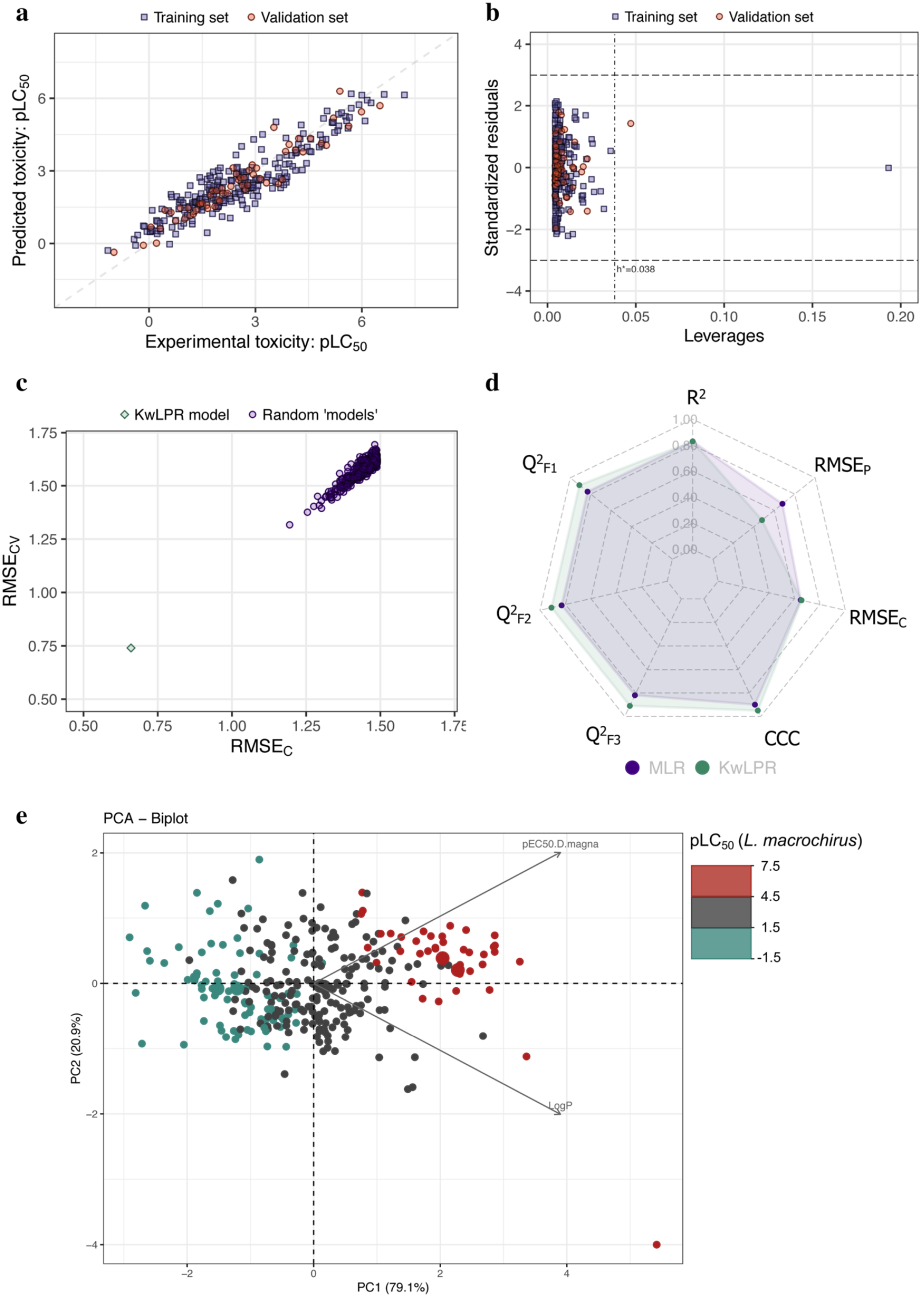
**a** Scatter plot of experimentally determined versus predicted values of pLC_50_. The formed straight line represents perfect agreement between the observed and calculated values. **b** Williams plot illustrating the applicability domain of the KwLPR model. **c** The results of the 500-fold Y-scrambling test. **d** Spider plot presenting the comparison of KwLPR model statistics with the MLR modeling approach. **e** PCA biplot

The complete dataset consists of 294 pesticides with quantitative toxicity value in the form of LC_50_ to *L. macrochirus* and *D. magna*. The toxicity values covered the toxicity range of 8.354 logarithmic units. In the original study, Basant et al. [20] employed the MLR approach for QSAAR model development, taking 235 pesticides as a training set and 59 as a validation set. By using two independent variables, namely the logarithmic value of the partition coefficient (Log P) and experimental toxicity value of *D. magna*, the authors successfully calibrated robust model for predicting the toxicity of pesticides to *L. macrochirus*. Both QSAAR models (i.e. MLR and KwLPR) reported the same value of correlation coefficient (R^2^ = 0.83). Whereas, all three external correlation validation metrics showed improved value for the KwLPR based QSAAR model (Q^2^_F1_ = 0.91; Q^2^_F2_ = 0.91; Q^2^_F3_ = 0.91 while previous one had 0.831, 0.831 and 0.818, respectively). A similar trend is observed for the CCC parameter where the present model reported a value of 0.95, and the earlier one was 0.90. The list of training/validation set chemicals along with the modelling parameters can be found in Supplementary information (Additional file 7: Table S2). The scatter plot (Fig. 6a) of experimental and predicted toxicity values illustrated that training and validation set chemicals scatter on both sides of the line of the perfect fit, and no points have deviated within ± 1 value. The Williams plot (Fig. 6b) for the applicability domain (AD) analysis suggested that one validation compound (Tributyltin oxide) is detected as an outlier. While one training compound showed a higher leverage value compare to the critical value (h^*^) of 0.035, which behaves as influential observations (X outlier) although not a response outlier (not Y outlier). We have also performed 500-fold Y-scrambling test where the plot in Fig. 6c suggested the KwPLR model was not obtained by chance and the model is extremely reliable. The Spider plot (Fig. 6d) suggested that KwPLR offered better validation outcomes over the MLR based model. Analysis of the PCA biplot graphically shown in Fig. 6e revealed that the toxicity of pesticides in *L. macrochirus* (pLC50) increases with increasing values of both related descriptors (i.e. LogP and pEC50 [mM] *D. magna*). Considering the vectors length and the angle between them it can be inferred that they are moderately positively correlated (r = 0.58) and paly an equally important role in determining toxicity to *L. macrochirus*.

### Case study 3

**Fig. 7:**
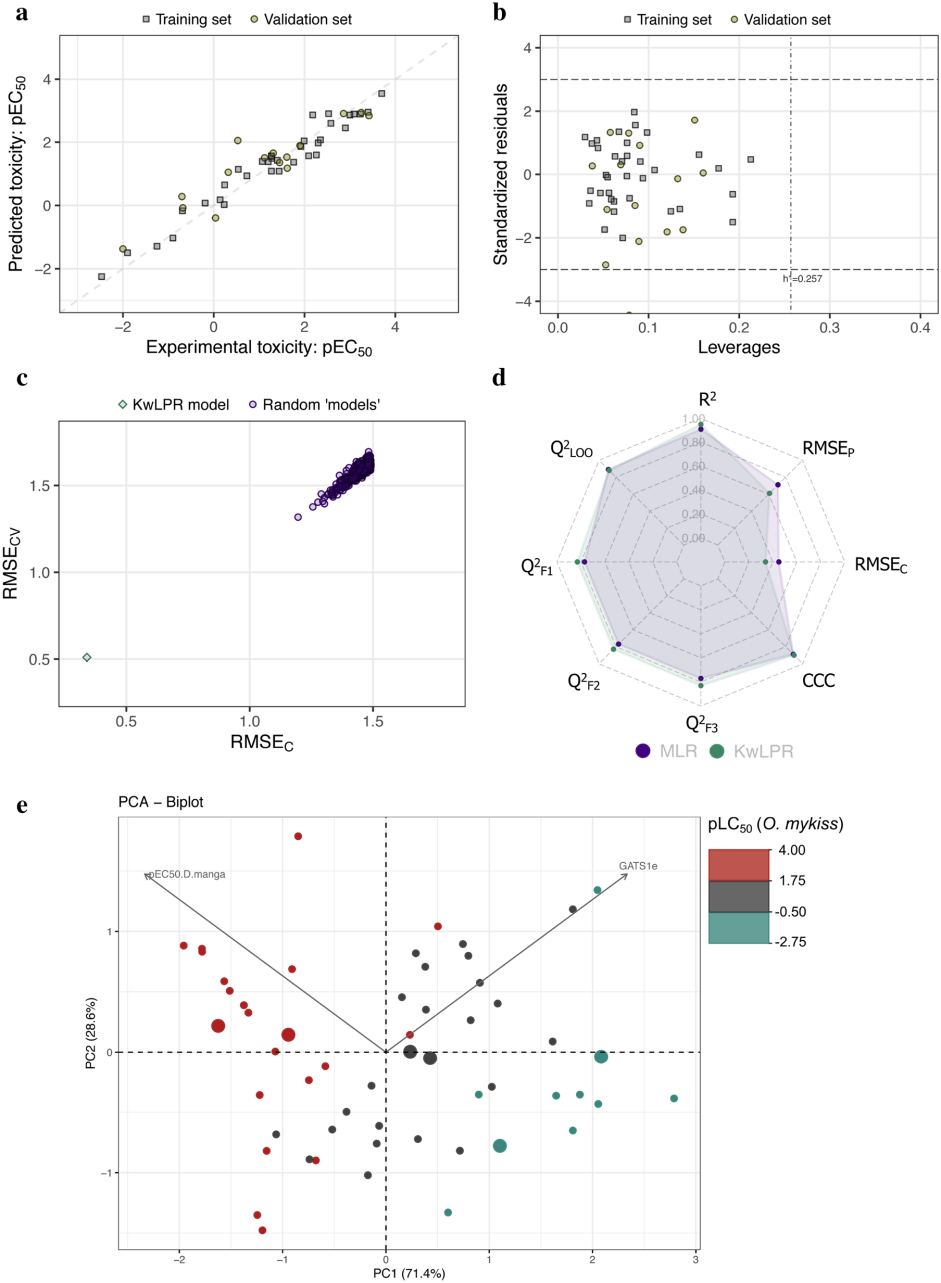
**a** Scatter plot of experimentally determined versus predicted values of pLC_50_. The formed straight line represents perfect agreement between the observed and calculated values. **b** Williams plot illustrating the applicability domain of the KwLPR model. **c** The results of the 500-fold Y-scrambling test. **d** Spider plot presenting the comparison of KwLPR model statistics with the MLR modeling approach. **e** PCA biplot

The dataset 3 includes toxicity value for *O. mykiss* and *D. magna* of 50 PPCPs as LC_50_. The modeled toxicity values of *O. mykiss* covered the toxicity range of 6.173 logarithmic units. The original model was developed using the MLR technique with 35 compounds in the training set and 15 in the validation set [21]. The present model uses *D. magna* toxicity and GATS1e as modeled descriptors like the previous model. The current KwLPR based QSAAR model slightly improved the R^2^ value (the present model reported a value of 0.95, and the earlier one was 0.91). A similar trend reported for external validation metrics. KwLPR based QSAAR model yielded considerably higher assessment statistics in the validation set (Q^2^_F1_ = 0.83; Q^2^_F2_ = 0.83; Q^2^_F3_ = 0.83), compared to the previous model (Q^2^_F1_ = 0.77; Q^2^_F2_ = 0.77; Q^2^_F3_ = 0.77). A sharp decline of RMSE_C_ and RMSE_P_ values is observed for the present model compared to the previous one, which is expected. It demonstrates the good predictive quality of the KwLPR derived model. The perfect scattering of training and validation set data points around the line's best fit in the scatter plot (Fig. 7a) illustrated the present model's predictive capability. The AD analysis suggested that all training and validation set compounds remain within the set boundaries of standardized residuals and critical leverage value, which implies that none of the PPCPs are outliers, and that their predictions are highly reliable. The Williams plot of AD analysis is illustrated in Fig. 7b. The 500-fold Y-scrambling test randomization plot (Fig. 7c) suggested that the developed KwPLR based model is highly reliable and not obtained by chance. Additionally, the spider plot (Fig. 7d) clearly showed similar internal and superior external regression-based validation metrics values for the KwLPR model compared to the previous MLR model. Considering error-based metrics, the KwLPR model also outperforms the MLR-based model. Analysis of the PCA biplot (Fig. 7e) confirmed an evident trend in the toxicity values of tested PPCPs. PC1 is positively correlated with the 2D autocorrelation descriptor, namely Geary autocorrelation of lag 1 weighted by Sanderson electronegativity (GATS1e) [21], and negatively correlated with the toxic to *D. magna* (pEC50). Thus, in general, low values of PC1 scores correspond to PPCPs with lower GATS1e and greater pEC50 (*D. magna*) resulted in higher toxicity to fish (*O. mykiss*). The angle between the loading vectors confirmed relatively low negative correlation (r = -− 0.43) among the variables. In turn, the similar length of vectors provided the evidence that both descriptors are equally important for the studied toxicity endpoint Supplementary information (Additional file 7: Table S3).

### Case study 4

**Fig. 8:**
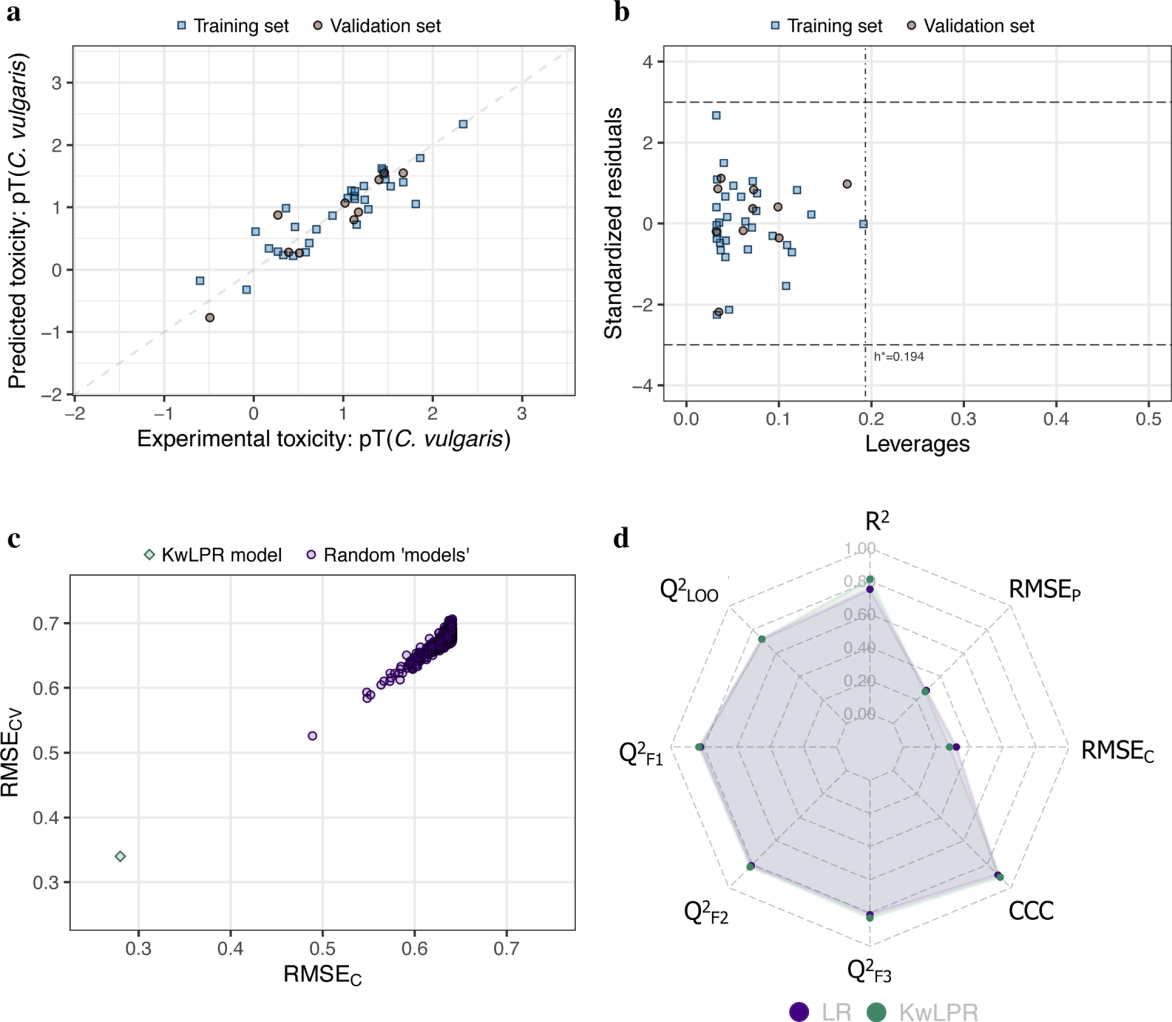
**a** Scatter plot of experimentally determined versus predicted values of pLC_50_. The formed straight line represents perfect agreement between the observed and calculated values. **b** Williams plot illustrating the applicability domain of the KwLPR model. **c** The results of the 500-fold Y-scrambling test. **d** Spider plot presenting the comparison of KwLPR model statistics with the LR modeling approach

This dataset includes the quantitative toxicity value of 41 substituted phenols to *Chlorella vulgaris* and *Tetrahymena pyriformis* expressed in pT = − logIGC_50_ (50% growth inhibition). The toxicity values of *C. vulgaris* (modeled toxicity) covered a range of 2.83 logarithmic units. The previous model was developed using simple linear regression (LR). In contrast, we have developed the present model using KwLPR with 31 training set substituted phenols, and the remaining ten compounds were considered for external validation purposes [22]. Like the earlier model, we have used only *T. pyriformis* acute toxicity as a modeled descriptor. The previous model reported a determination coefficient (R^2^) of 0.75, while the present model using KwLPR increases this value to 0.81 using the same division as well as modeled descriptor. While, all external correlation validation metrics showed slightly improved value for the KwLPR based QSAAR model (Q^2^_F1_ = 0.83; Q^2^_F2_ = 0.82; Q^2^_F3_ = 0.83 while previous one range from 0.81 ÷ 0.82). Similarly, in the case of error-based metrics, lower values for RMSE_C_ and RMSE_P_ were obtained for the present model compare to the previous, which is the indication of better predictive nature of the KwLPR model (0.28 and 0.27, respectively) over the LR model (0.32 and 0.28, respectively). The scatter plot (Fig. 8a) revealed a good correlation between the experimental and predicted toxicity. The resulting graph showed that most points were close and scattered around both sides to fit the best line. Even the most scattered point showed the residual error of less than one numerical value, implicating the good quality of the developed model. The Williams plot (Fig. 8b) suggested that neither training nor validation set compounds are identified as X or Y-outliers, and their predictions are highly reliable and acceptable. The 500-fold Y-scrambling test supports the robustness of the KwPLR model (Fig. 8c). The spider plot (Fig. 8d) clearly illustrated almost similar metrics values for Q^2^_LOO_, Q^2^_F1_, Q^2^_F2_, Q^2^_F3_, CCC, and RMSE_P_ while the KwLPR model outperforms the LR model in case of better results for R^2^ and RMSE_C_ metrics. In this particular case, since only one independent variable was used to develop the KwLPR model, the interrelationship among the modeled toxicity and the related descriptor can be assessed through the correlation coefficient. Thus, in general, strong positive correlation (r = 0.88) indicates that substituted phenols highly toxic to *T. pyriformis* are also highly toxic to *C. vulgaris* Supplementary information (Additional file 7: Table S4).

### Case study 5

**Fig. 9:**
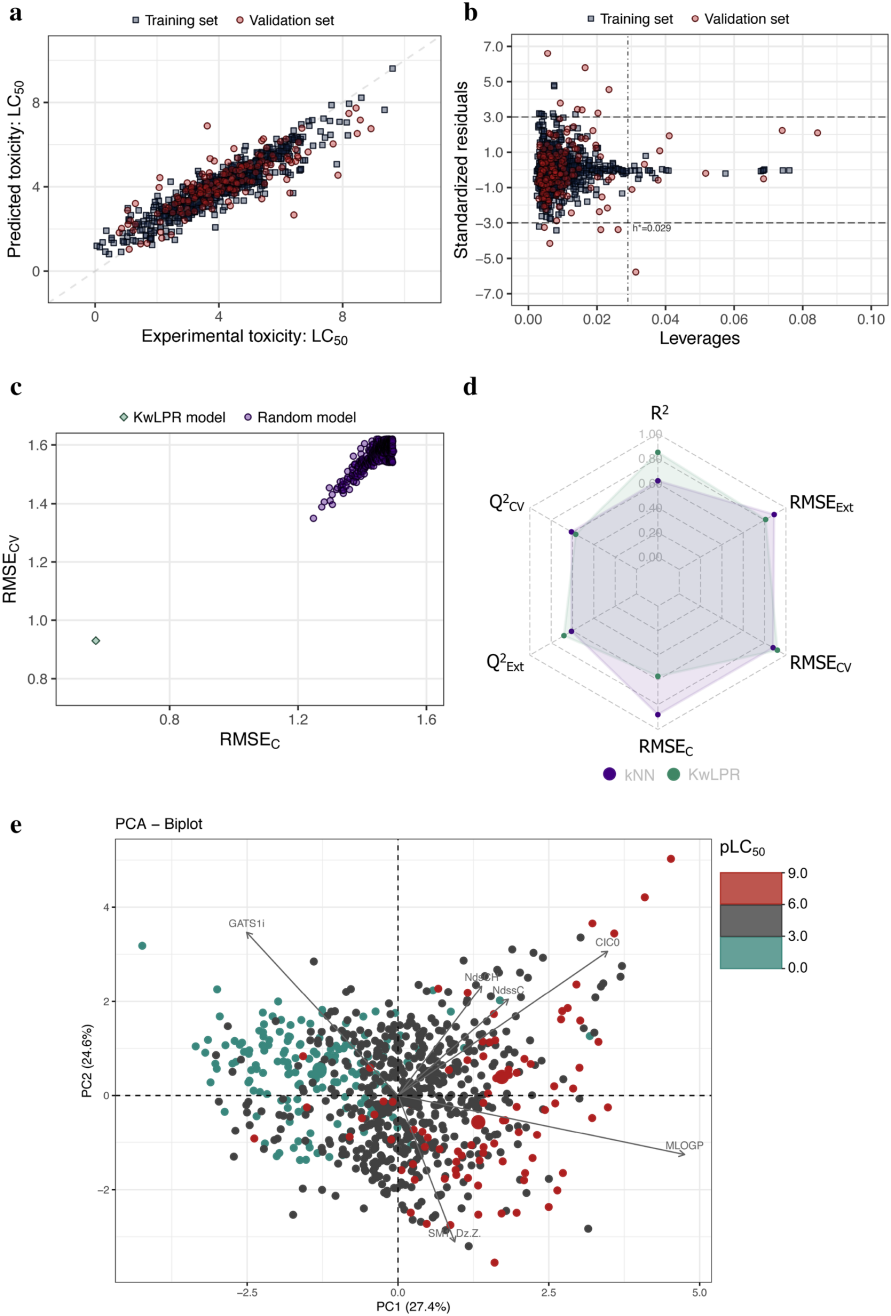
**a** Scatter plot of experimentally determined versus predicted values of pLC_50_. The formed straight line represents perfect agreement between the observed and calculated values. **b** Williams plot illustrating the applicability domain of the KwLPR model. **c** The results of the 500-fold Y-scrambling test. **d** Spider plot presenting the comparison of KwLPR model statistics with the LR modeling approach. **e** PCA biplot

The acute toxicity (LC_50_ 96 h) of 908 diverse organic chemicals towards the fathead minnow (*Pimephales promelas*) were considered for the last case study [23]. Cassotti et al. [23] employed non-linear *k*NN approach to develop the predictive QSAR model and they obtained a model with *k* = 6 (R^2^ = 0.62; Q^2^_cv_ = 0.61; Q^2^_Ext_ = 0.61). The studied toxicity range is: 0.053 to 9.612. The calibration and validation of the KwPLR model was carried out using the same training/test set (n_T_ = 726/n_V_ = 182) and explanatory variables as provided by the authors of the original work. The list of training/validation set chemicals along with the modelling parameters can be found in Supplementary information (Additional file 7: Table S5). The KwPLR model reported around 37% improvement of quality of the model fit over *k*NN model. Although, the model’s cross-validation for the *k*NN model showed higher value than the KwLPR model, the better prediction was achieved for the validation set, which can be confirmed by Q^2^_EXT_ parameter (0.61 and 0.68 for *k*NN and KwLPR model, respectively). The perfect scattering of training and validation set data points around the line's best fit in the scatter plot (Fig. 9a) demonstrated the present model's predictive capability. The Williams plot of AD analysis is reported in Fig. 9b. The 500-fold Y-scrambling test randomization plot (Fig. 9c) suggested that the developed KwPLR based model is highly reliable and not obtained by chance. Additionally, the spider plot (Fig. 9d) clearly showed that except internal cross-validation (Q^2^_CV_) metric, all remaining validation metrics values for the KwLPR model compared to the previous *k*NN model are superior in terms of accepted threshold. Interpretation of PCA biplot (Fig. 9e) revealed that out of six descriptors employed for the KwLPR model development the most influential variables (indicated by the longest arrow vectors) were Moriguchi octanol–water partitioning coefficient (MLOGP), Complementary Information Content index (CIC0) that accounts for the presence of heteroatoms, and the 2D autocorrelation descriptor that considers the ionization potential of bonded atoms (GATS1i). Two of these variables (*i.e.* MLOGP and CIC0) were positively correlated, whereas the last (*i.e.* GATS1i) was negatively correlated. In light of these it can be stated, that highly lipophilic chemicals with large CIC0 and low GATS1i are characterized by an inherently greater toxicity towards *P. promelas* compared to chemicals with lower MLOGP and CIC0 and larger GATS1i.

The details of the factors that influence the modeling outcomes (i.e., bandwidth method selection, bandwidth parameters, local polynomial's degree, kernel function) as well as internal and external validation metrics of all five KwLPR based QSAAR models are given in Table 2. If one compares the accessible validation metrics of the previously developed model and present ones (Tables 1 and 2), it is quite clear that for all five case studies, the quality of current models is superior.

**Table 2:** The details of the present KwLPR based QSAR/QSAAR models

Case study	Bandwidth method selection		Bandwidth					Local polynomial's degree	Kernel function
Case study 1	Least Squares Cross-Validation Method		LogP			0.410		0(Constant)	Gaussian
			pEC_50_ (mM) (*D. magna*)			0.213			
	n_T_ = 254; R^2^ = 0.85; RMSE_C_ = 0.60; Q^2^_LOO_ = 0.79; RMSE_CV_ = 0.70								
	n_V_ = 64; Q^2^_F1_ = 0.88; Q^2^_F2_ = 0.88; Q^2^_F3_ = 0.88; CCC = 0.93; RMSE_P_ = 0.54								
Case study 2	Expected Kullback–Leibler cross-validation Method		LogP			0.416		0 (Constant)	Gaussian
			pEC_50_ (*D. magna*)			0.292			
	n_T_ = 235; R^2^ = 0.83; RMSE_C_ = 0.66; Q^2^_LOO_ = 0.79; RMSE_CV_ = 0.74								
	n_V_ = 59; Q^2^_F1_ = 0.91; Q^2^_F2_ = 0.91; Q^2^_F3_ = 0.91; CCC = 0.95; RMSE_P_ = 0.48								
Case study 3	Least Squares Cross-Validation Method		pEC_50_ (*D. magna*)			0.357		0 (Constant)	Gaussian
			GATS1e			0.585			
	n_T_ = 35; R^2^ = 0.95; RMSE_C_ = 0.34; Q^2^_LOO_ = 0.88; RMSE_CV_ = 0.51 n_V_ = 15; Q^2^_F1_ = 0.83; Q^2^_F2_ = 0.83; Q^2^_F3_ = 0.83; CCC = 0.90; RMSE_P_ = 0.61								
Case study 4	Direct Plug-in Method			pT (*T. pyriformis*)	0.399		1 (Local linear)		Gaussian
	n_T_ = 31; R^2^ = 0.81; RMSE_C_ = 0.28; Q^2^_LOO_ = 0.72; RMSE_CV_ = 0.34 n_V_ = 10; Q^2^_F1_ = 0.83; Q^2^_F2_ = 0.82; Q^2^_F3_ = 0.83; CCC = 0.91; RMSE_P_ = 0.27								
Case study 5	Least Squares Cross-Validation Method			MLOGP	0.417		0 (Constant)		Gaussian
				CIC0	0.584				
				SM1_Dz(Z)	0.512				
				GATS1i	0.535				
				NdsCH	0.781				
				NdssC	0.521				
	n_T_ = 726; R^2^ = 0.85; RMSE_C_ = 0.57; Q^2^_CV_ = 0.57; RMSE_CV_ = 0.93 n_V_ = 182; Q^2^_EXT_ = 0.68; RMSE_EXT_ = 0.87; CCC = 0.79								

**Fig. 10:**
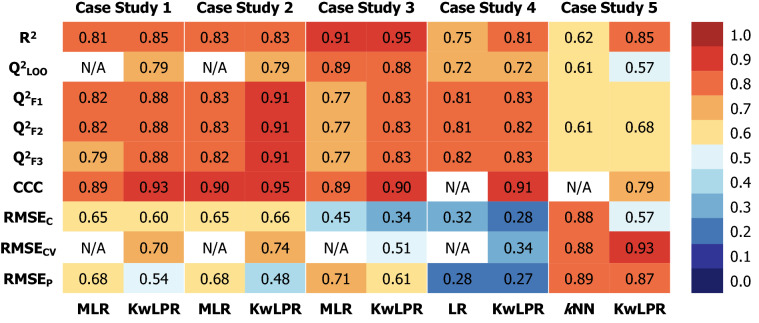
Comparison of validation metrics for all five case studies employing linear and non-linear regression technique over the KwLPR

Finally, to make the comparison between the proposed KwLPR approach and the traditional linear and non-linear regression-based techniques more meaningful, we have considered three error-based metrics RMSE_C_ (Root Mean Square Error of Calibration), RMSE_CV_ (Root Mean Square Error of Cross-Validation) for the training set and RMSE_P_ (Root Mean Square Error of Prediction) for validation set along with six classical internal and external regression-based metrics (Fig. 10). Four new QSAR/QSAAR models achieved higher (Case studies 1, 3, 4 and 5) and one model (Case study 2) achieved identical R^2^ value in the case of the KwLPR model compared to linear and non-linear models. While comparing other regression-based metrics values, in all cases, KwLPR models outperformed the linear as well as non-linear models. As no previous QSAAR studies considered RMSE_CV_, we can't compare their results with the present models, but all the values obtained here are acceptable. Lower RMSE_C_ and RMSE_P_ values are required to show the KwLPR approach's superiority over the traditional linear and non-linear regression approaches. The RMSE_C_ value of case study 2 is better for the linear model compared to KwLPR. Except for this one instance, both error-based metrics demonstrated improved outcomes for all remaining three case studies (1, 3 and 4) in the KwLPR model's case over the linear models. Similarly, the KwLPR model for case study 5 improve the quality of the model fit (R^2^) by over 37% compare to non-linear *k*NN based model.

The reason behind the better statistically predictive model for all five cases is that the regression coefficients of the KwLPR are estimated with a sliding smoothing window by fitting a polynomial of degree (0 or 1) locally at each query point using specific bandwidth method selection (direct plug-in method; least squares cross-validation method or expected Kullback–Leibler cross-validation method) and kernel function (Gaussian) which help in minimizing the MSE of the individual model. Thus, for the precise prediction of external test compounds (new or query compounds), especially for biological activity and toxicity of chemicals and pharmaceuticals, the KwPLR method is a superior choice over the traditional linear and non-linear regression methods for the development of QSAR/QSAAR models. On the other hand, even though the KwLPR approach has several advantages, it has certain constraints. One is related to the interpretability of the model. The interpretation of a kernel-weighted local polynomial regression model requires an external technique (here PCA) to provide explanations for an existing model (so-called post-hoc interpretability). Hence, one should be aware that the interpretability of a KwLPR model, as several other widely-used nonlinear models (e.g. *k*NN, RF, SVM) is a necessary trade-off of its improved predictive accuracy [44, 45].

## Conclusions

Based on comparison with five diverse databases the present work demonstrates the effectiveness and practicability of the KwLPR approach to develop QSAR/QSAAR models. We also demonstrate its advantages compared to the traditional linear and non-linear regression-based techniques. Irrespective of database size and chemical diversity of compounds, using the same training and validation sets and modeled descriptors; the KwLPR model offers lower residual errors for both training and validation sets than the other evaluated approaches accomplishing the primary aim of the QSAR/QSAAR models. The KwLPR are estimated with a sliding smoothing window by fitting a polynomial of degree (0 or 1) locally at each query point using specific bandwidth and kernel functions which help in minimizing the MSE of the individual model and chemicals. It is characterized by mathematical simplicity and interpretability of the classical least squares method with the flexibility of nonlinear regression. Thus, the KwLPR approach can be applied to develop QSAR/QSAAR model avoiding the disadvantages of traditional linear regression approaches. This is facilitated by availability to all users of our freely accessible *KwLPR.RMD* script in R programming language.

## Supplementary Information

**Additional file 1.** Input data for Case study-1.

**Additional file 2.** Input data for Case study-2.

**Additional file 3.** Input data for Case study-3.

**Additional file 4.** Input data for Case study-4.

**Additional file 5.** Input data for Case study-5.

**Additional file 6.** KwLPR.RMD script.

**Additional file 7.** Overview of KwLPR modelling results.
